# Mitochondrial pathway is involved in the protective effects of alpha-ketoglutarate on hydrogen peroxide induced damage to intestinal cells

**DOI:** 10.18632/oncotarget.20426

**Published:** 2017-08-24

**Authors:** Qian Jiang, Gang Liu, Xiuqi Wang, Yongqing Hou, Yehui Duan, Guoyao Wu, Yulong Yin, Kang Yao

**Affiliations:** ^1^ Key Laboratory of Agro-ecological Processes in Subtropical Region, Institute of Subtropical Agriculture, Chinese Academy of Sciences; Hunan Provincial Engineering Research Center for Healthy Livestock and Poultry Production; Scientific Observing and Experimental Station of Animal Nutrition and Feed Science in South-Central, Ministry of Agriculture, Changsha 410125, China; ^2^ University of Chinese Academy of Sciences, Beijing 100039, China; ^3^ Hunan Collaborative Innovation Center for Utilization of Botanical Functional Ingredients; Hunan Collaborative Innovation Center of Animal Production Safety, Changsha 410128, China; ^4^ College of Animal Science, South China Agricultural University/National Engineering Research Center for Breeding Swine Industry, Guangzhou 510642, Guangdong Province, China; ^5^ Hubei Key Laboratory of Animal Nutrition and Feed Science, Wuhan Polytechnic University, 430023 Wuhan, China; ^6^ Department of Animal Science, Texas A&M University, College Station, TX 77843, USA; ^7^ Hubei Collaborative Innovation Center for Animal Nutrition and Feed Safety, Wuhan Polytechnic University, Wuhan 430023, China; ^8^ College of Animal Science and Technology, Hunan Agricultural University, Changsha 410128, China

**Keywords:** alpha-ketoglutarate, oxidative damage, cell apoptosis, mitochondrial respiration, intestinal porcine epithelial cells

## Abstract

Alpha-ketoglutarate, a key intermediate in the Krebs cycle, has been reported to benefit intestinal health. We tested whether alpha-ketoglutarate can protect intestinal cells against hydrogen peroxide induced damage and aimed to reveal the underlying mechanism. Intestinal porcine epithelial cell line J2 were cultured in Dulbecco’s Modified Eagle Medium-High glucose with or without alpha-ketoglutarate and hydrogen peroxide. Cell viability, proliferation, mitochondrial respiration, mitochondrial membrane potential, antioxidant function, apoptosis and mitochondrial-dependent apoptotic pathways were determined. Our experiments demonstrated that, first, exposure to 100μM hydrogen peroxide decreased cell viability, DNA synthesis, mitochondrial respiration and antioxidant function, and increased apoptosis. Second, 2mM alpha-ketoglutarate addition attenuated hydrogen peroxide-induced cell cycle arrest, and improved cell viability, DNA synthesis, mitochondrial respiration and antioxidant function. Third, alpha-ketoglutarate enhanced tricarboxylic acid cycle activity, mitochondrial respiration, and decrease the intracellular content of reactive oxygen species. Finally, alpha-ketoglutarate stabilized the mitochondrial membrane potential, increased the ratio of Bcl-2/Bax, decreased the release of cytochrome c and activation of caspase-3, thereby prevented cell apoptosis. Altogether, we proposed that alpha-ketoglutarate protects intestinal cells against hydrogen peroxide-induced damage partly via mitochondria dependent pathway.

## INTRODUCTION

The intestinal epithelium is the most important internal barrier against the invasion of pathogenic bacteria [1], and functions as an important part of the first line of defense [2]. In swine production, weaning stress has been demonstrated to be associated with gastrointestinal disorders such as changes in intestinal barrier function and absorption, leading to diarrhea and increased disease susceptibility [3, 4]. In addition, a recent study indicated that weaning can induce oxidative stress [5]. Accordingly, one mechanism linking weaning stress to intestinal disease may be oxidative stress. Oxidative stress refers to the imbalance of oxidation and antioxidation, which leading to accumulation of oxidation intermediates including superoxide ion (O^2-^), hydroxyl radical (OH) and H_2_O_2_ [6]. It causes intestinal disorders possibly via [2, 7, 8] : 1) disrupting the structures of the intestinal barrier, 2) influencing mitosis or apoptosis of intestinal epithelial cells, or 3) distorting the differentiation potential of epithelial cells. Therefore, maintaining a balanced redox status is essential for ensuring intestinal health [9].

Alpha-ketoglutarate (AKG) is a gut nutrient and may be used directly in the intestinal mucosa as an energy resource without elevating the nitrogen load [10, 11]. Dietary supplementation of 1% AKG has been found to alleviate mucosal damage and improve the absorptive function of the small intestine in the endotoxin-challenged piglets [12]. In addition, AKG is an intermediate in the tricarboxylic acid (TCA) cycle, and can be transformed to succinyl-CoA by AKG dehydrogenase. AKG dehydrogenase is sensitive to ROS, and inhibition of this enzyme induced by oxidative stress may be malignant to the normal metabolism of mitochondria [13]. In addition, AKG is a natural ubiquitous collector of amino groups in tissues, and is thus, a potent detoxifying agent [14]. For instance, AKG can function as an antidote to cyanide poisoning, wherein it reacts with cyanide molecules to form cyanohydrin, a reaction product [15]. In this regard, it is far more powerful than natural antioxidants such as vitamin C [14]. Thus, AKG may function as a strong ammonia- and phosphate-binding factor and indirectly stabilize redox state in organisms [11]. In support of this view, previous studies show that administration of AKG can improve serum redox homeostasis, thus conferring protection to arteries from vascular damage caused by free radicals [16]. Other studies have also demonstrated the antioxidative effects of AKG in cultured hepatoblastoma cells [17, 18]. These interesting observations concerning the effects of AKG on intestinal disease and oxidative stress prompted us to further investigate the effects of AKG on oxidative stress-induced intestinal damage *in vitro*, with an emphasis on identifying the possible mechanisms involved.

Intestinal porcine epithelial cell lines including IPEC-1, IPI-2I and IPEC-J2 are commonly used in the past research [19]. IPI-2I is a plasmid transformed cell line isolated from the ileum, whereas IPEC-1 and IPEC-J2 are normal cell lines, and IPEC-1 cell line was isolated from a mixture of ileal and jejunal tissue, while IPEC-J2 cell line was isolated from the jejunum [20, 21]. In the present study, oxidative stress was induced by hydrogen peroxide (H_2_O_2_), the IPEC-J2 cell line was used as the *in vitro* model of the porcine intestinal epithelium and to investigate the effects of AKG on the oxidative stress responsive intestine, aiming to reveal the mechanisms underlying the regeneration and repair of the small-intestinal mucosa. Compared with transformed cell lines, two major advantages favor IPEC-J2 cells as *in vitro* model of intestine: 1) differentiative potential and proliferation profiles are similar to primary intestinal epithelial cells, and 2) some glycocalyx-bound mucus proteins, cytokines, chemokine, and display Toll-like receptors can be regularly produced or activated, highly resembling the *in vivo* milieu and modeling the gastrointestinal tract [21–24]. Our results strongly indicate a protective role of AKG against H_2_O_2_-induced enterocyte damage via improved mitochondrial function and mitochondrial-dependent pathway.

## RESULTS

### Effects of H_2_O_2_ and AKG on the viability of IPEC-J2 cells

**Figure 1:**
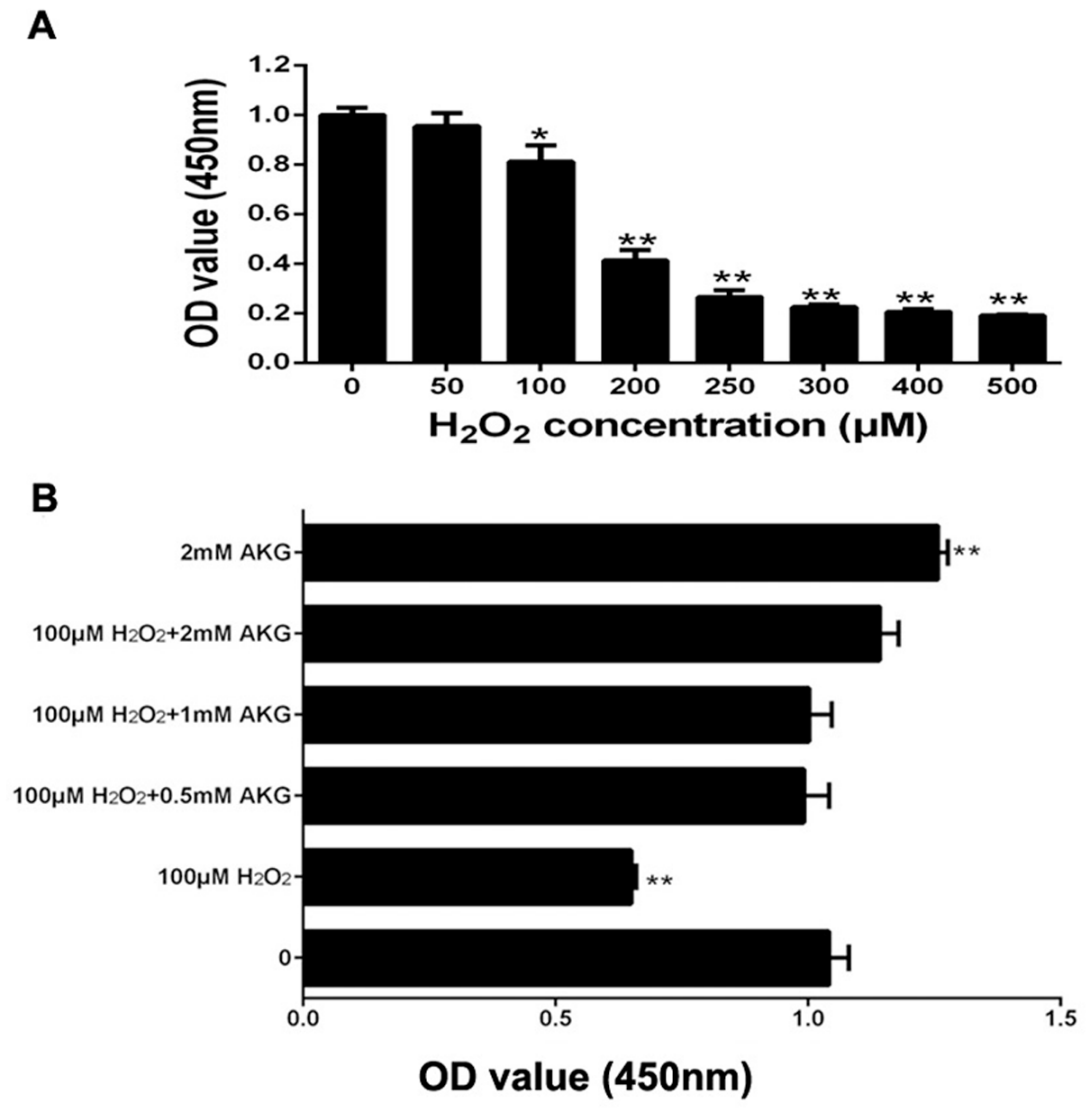
Cell proliferation in IPEC-J2 cells **(A)** Effects of increasing concentrations of H_2_O_2_ for 4h on cell proliferation; **(B)** Effects of addition of increasing concentrations of AKG from 0 to 2 mM to 100 μM H_2_O_2_ for 2 days on cell proliferation. Cell viability was quantified by CCK-8 assay. Data are expressed as means ± SEM of at least three independent experiments. *P < 0.05 and **P < 0.01.

**Figure 2:**
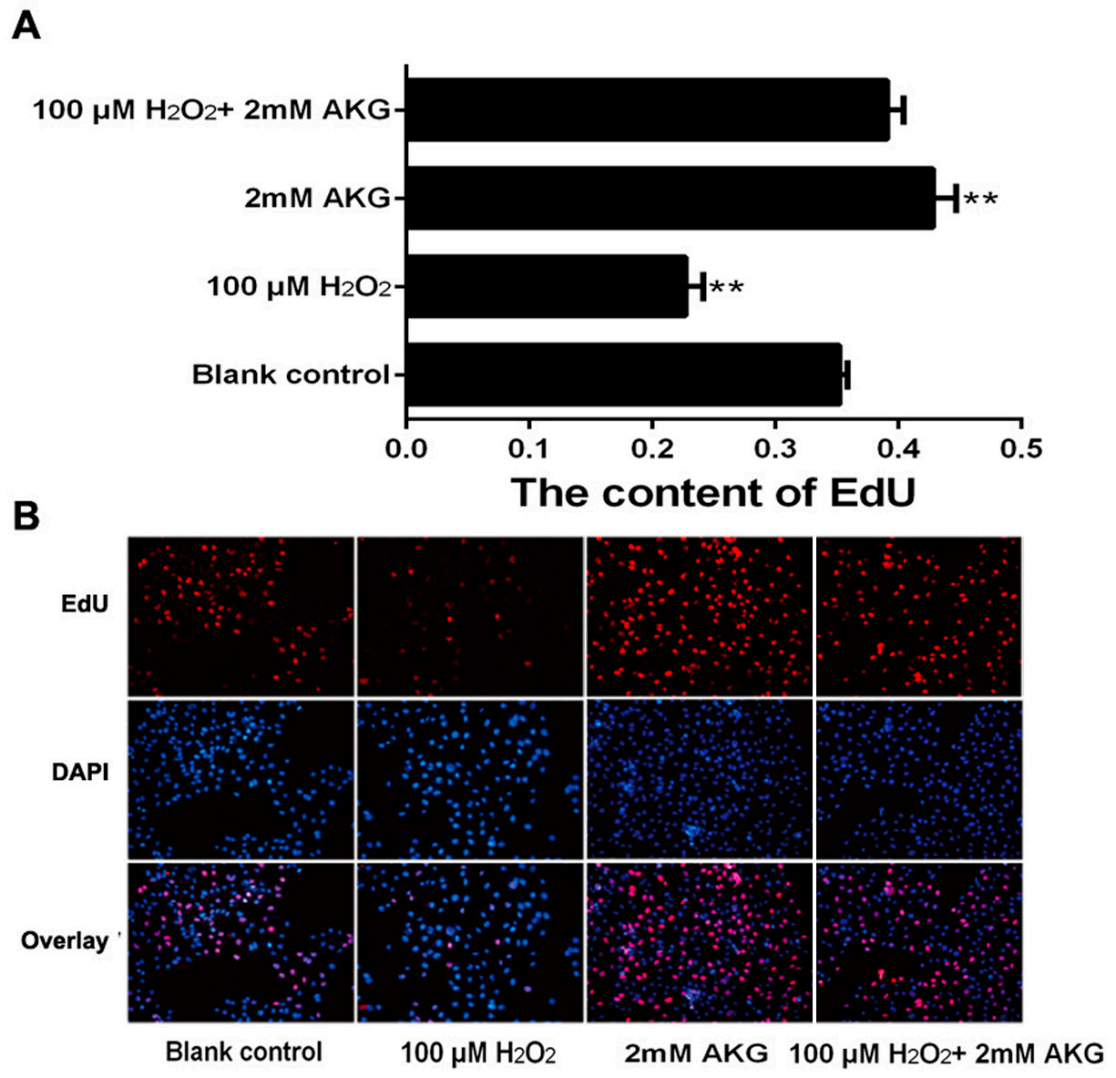
DNA synthesis in IPEC-J2 cells DNA synthesis during the proliferation of IPEC-J2 cells was quantified by EdU incorporation (red color) using Cell-Light™ EdU Kit (Rui Bo Biotechnology Limited Company, Guangzhou, China). Nuclei are shown in blue color. Cells were treated without (Blank control) or with 100 μM H_2_O_2_, 2 mM AKG, or 100 μM H_2_O_2_ plus 2 mM AKG, respectively. **(A)** The percentage of EdU-positive cells (the number of red nuclei versus the number of blue nuclei in at least five different microscopic fields of vision). **(B)** Representative images of EdU staining (magnification × 200) of cells. Data are expressed as means ± SEM of at least three independent experiments. *P < 0.05 and **P < 0.01.

The viability assay of IPEC-J2 cells was performed by first treating the cells with different concentrations of H_2_O_2_ (0, 50, 100, 200, 250, 300, 400, or 500 μM) for 4 h. The results indicated that H_2_O_2_ decreased IPEC-J2 cell viability in a dose-dependent manner; and when itsconcentration was increased to 100 μM, H_2_O_2_ showed significant inhibitory effects on cell viability (*P* < 0.05) (Figure 1A). We then chose this concentration of H_2_O_2_ (100 μM) for further experiments. To examine the effects of AKG on the viability of H_2_O_2_-treated IPEC-J2 cells, different concentrations of AKG (0, 0.5, 1, or 2 mM) were added to the cells pretreated with 100 μM H_2_O_2_. Additionally, IPEC-J2 cells were treated without AKG (blank control) or with 2 mM AKG (positive control), without being subjected to oxidative stress (100 μM H_2_O_2_). AKG enhanced the viability of H_2_O_2_-pretreated IPEC-J2 cells in a dose-dependent manner, and the combination of 2 mM AKG + 100 μM H_2_O_2_ showed the maximum effect in comparison with the positive control (*P* < 0.05) (Figure 1B). Additionally, we determined the content of EdU in IPEC-J2 cells, as illustrated in Figure 2. Our results showed that the percentages of EdU-positive cells decreased in response to H_2_O_2_ treatment (*P* < 0.05) (Figure 2A). However, the addition of AKG (2 mM) to cells pretreated with 100 μM H_2_O_2_ resulted in an increase in the number of EdU-positive cells. Moreover, the EdU content was the highest in cells treated only with AKG (2 mM) (*P* < 0.05). Based on these results, we employed 100 μM H_2_O_2_ and 2 mM AKG in further experiments.

### Cell cycle arrest and apoptosis

**Figure 3:**
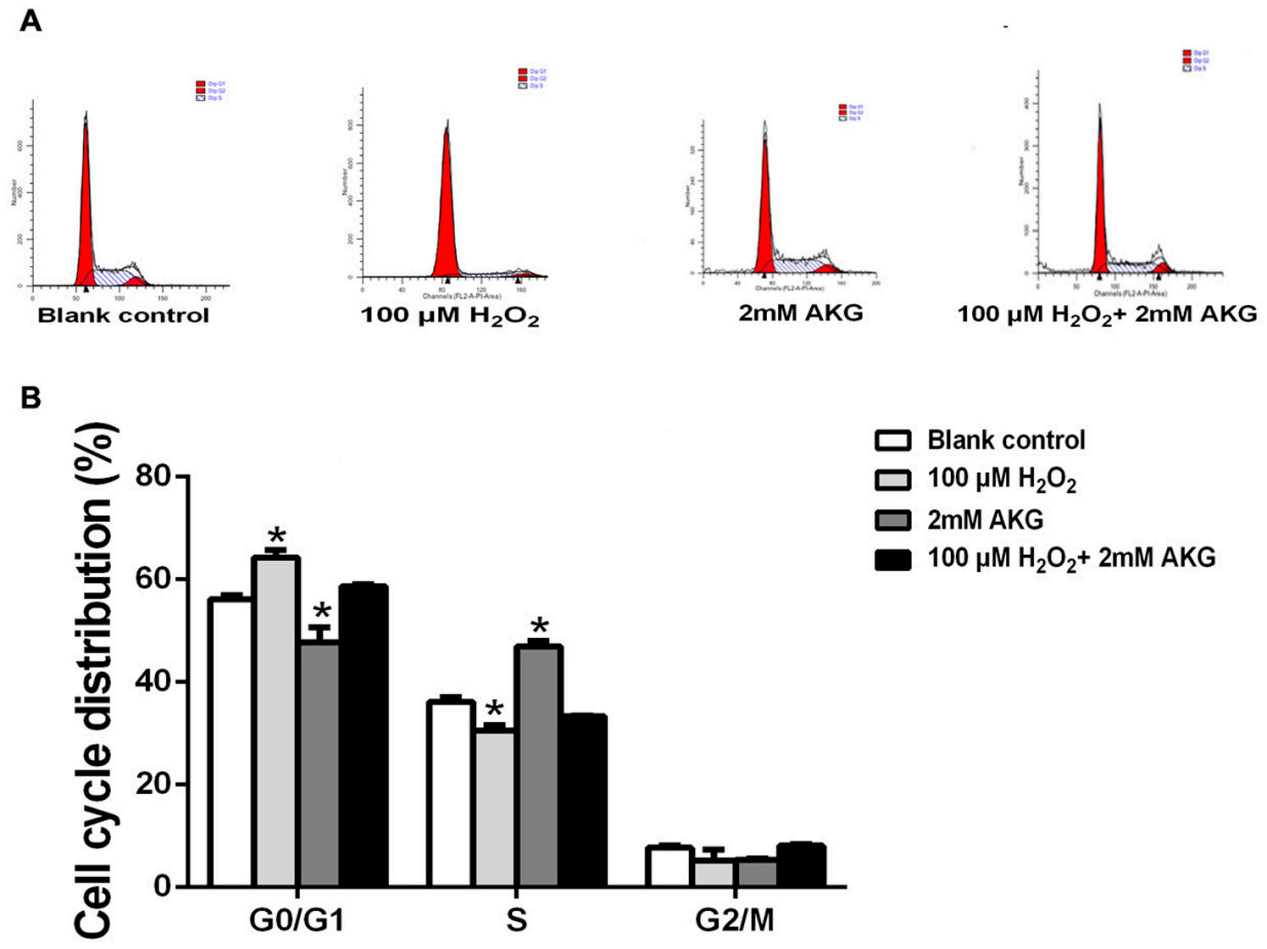
Cell cycle of IPEC-J2 cells analyzed using flow cytometry **(A)** Cells were treated without (Blank control) or with 100 μM H_2_O_2_, 2 mM AKG, or 100 μM H_2_O_2_ plus 2 mM AKG, respectively. Then, cells were analyzed by flow cytometry for the cell cycle. G0/G1, S, G2/M phase cells presented as percentages. **(B)** Data are expressed as means ± SEM of at least three independent experiments. *P < 0.05 and **P < 0.01.

**Figure 4:**
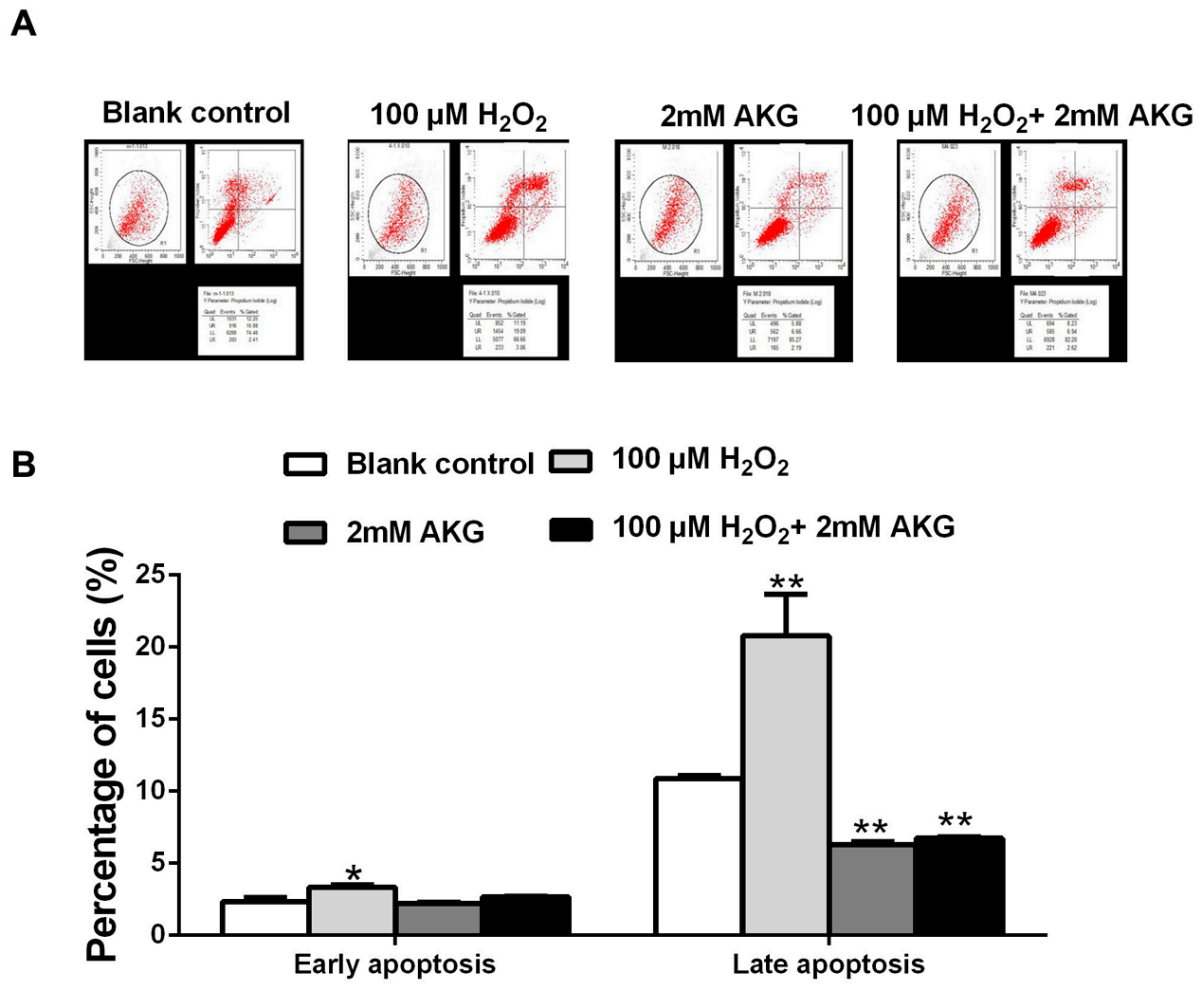
Cell apoptosis in the IPEC-J2 cells **(A)** Cells were treated without (Blank control) or with 100 μM H_2_O_2_, 2 mM AKG, or 100 μM H_2_O_2_ plus 2 mM AKG, respectively. Then, cells were analyzed by flow cytometry for the cell apoptosis. **(B)** Data are expressed as means ± SEM of at least three independent experiments. *P < 0.05 and **P < 0.01.

Flow cytometry analysis was performed to provide further evidence that inhibition of cell cycle progression by H_2_O_2_ was responsible for its anti-cell proliferative effect, and that AKG could attenuate this effect of H_2_O_2_. IPEC-J2 cells were treated without H_2_O_2_ (blank control) or with H_2_O_2_ (100 μM), AKG (2 mM), and H_2_O_2_ (100 μM) + AKG (2 mM). As shown in Figure 3, cell cycle distribution patterns of IPEC-J2 differed in response to different treatments. When compared with the blank control, 100 μM H_2_O_2_ induced a significant G0/G1 cell cycle arrest along with decreased number of cells in S phase (*P* < 0.05). In contrast, 2 mM AKG significantly decreased the cell cycle arrest at the G0/G1 phase and increased the number of cells in S phase (*P* < 0.05). As expected, the addition of 2 mM AKG to IPEC-J2 cells pretreated with 100 μM H_2_O_2_ resulted in a decrease in the number of cells in G0/G1 phase and an increase in the number of cells in S phase. However, no difference was observed in the G2/M phase cells among the groups (Figure 3B). Additionally, cell apoptosis was analyzed by Annexin V-FITC/PI staining. The results showed that when compared with the blank control group, 100 μM H_2_O_2_ increased the percentages of both early and late apoptotic cells (*P* < 0.05), while the addition of 2 mM AKG alone or with 100 μM H_2_O_2_ to cells decreased the above parameters (*P* < 0.05) (Figure 4).

### Intracellular ROS levels

**Figure 5:**
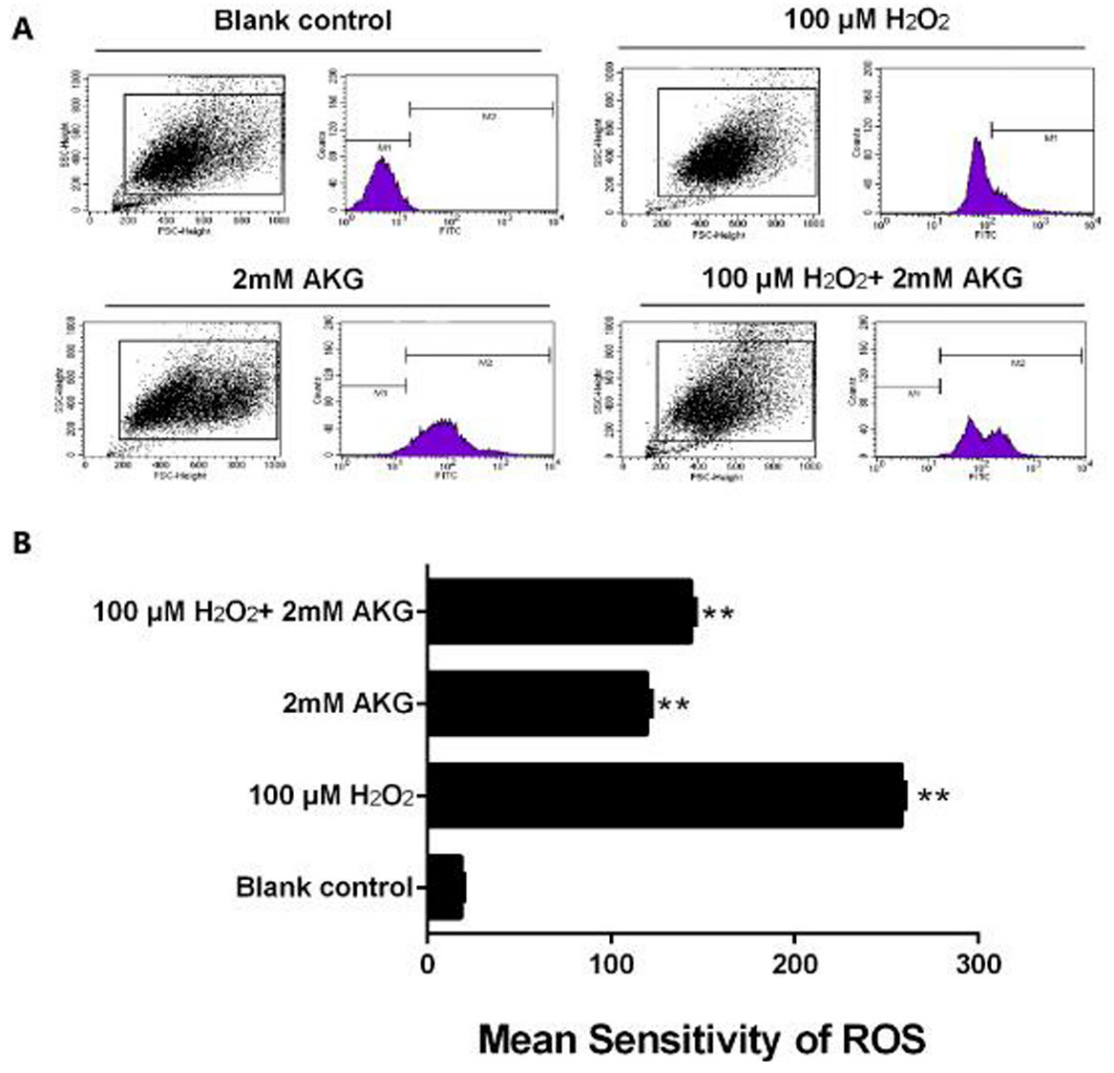
ROS content in IPEC-J2 cells **(A)** Graphical representation of ROS content analyzed using flow cytometry. **(B)** Representation of analyzed mean sensitivity of ROS. Data are expressed as means ± SEM of at least three independent experiments. *P < 0.05 and **P < 0.01.

Flow cytometry technique was employed to evaluate the alteration of intracellular or mitochondrial ROS in the treated IPEC-J2 cells. As presented in Figure 5A, B, H_2_O_2_-induced oxidative stress significantly enhanced the ROS content of the cells (*P* < 0.05). However, the addition of 2 mM AKG to cells pretreated with 100 μM H_2_O_2_ reduced the ROS overload, although it was still higher than in the control group (*P* < 0.05).

### Mitochondrial bioenergetics

**Figure 6:**
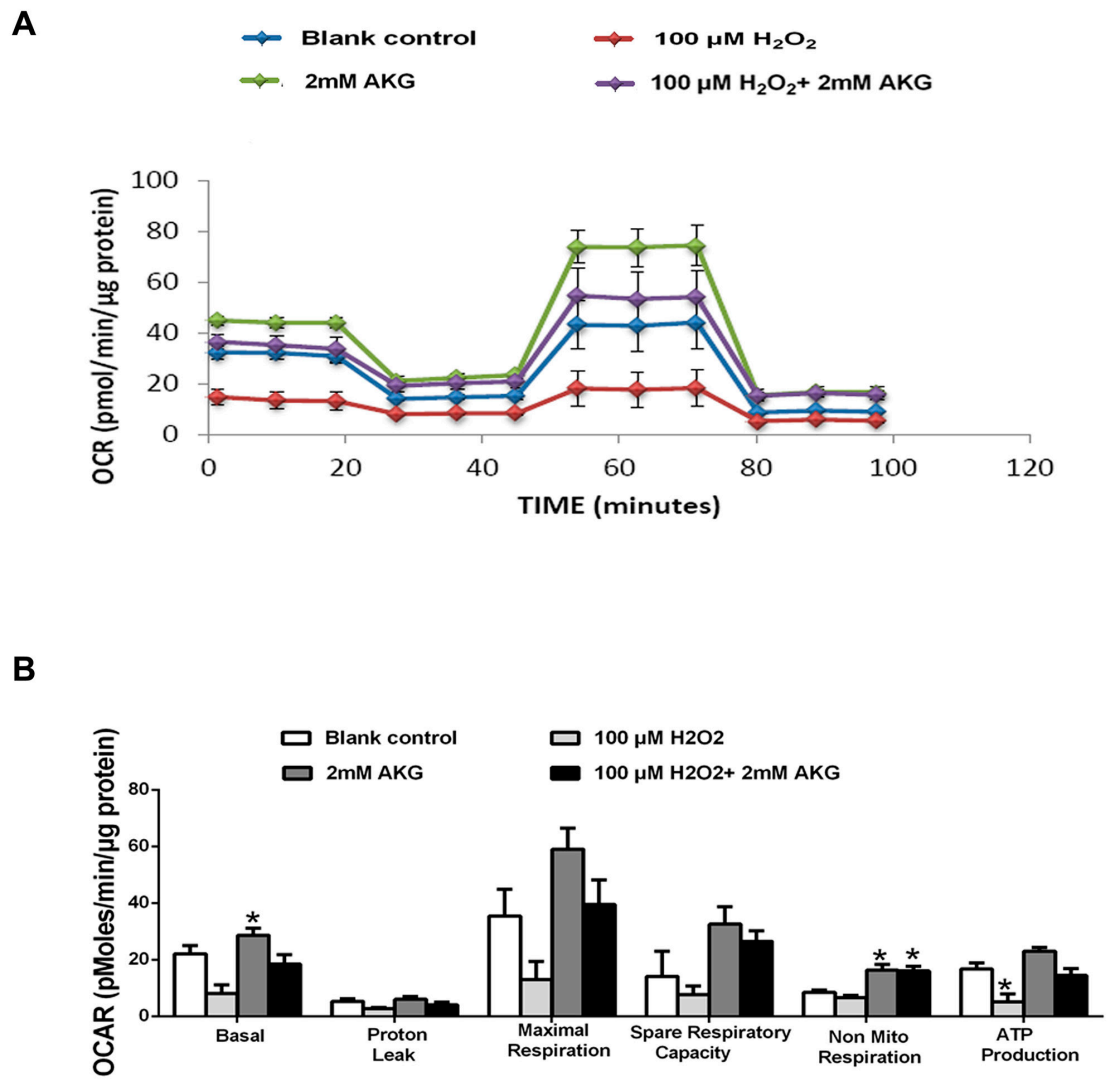
Mitochondrial respiration of IPEC-J2 cells Schematic **(A)** and oxygen consumption rate (OCR) **(B)** assessed by extracellular flux analysis. OCR was measured under basal conditions followed by the sequential addition of oligomycin (0.5 μM), FCCP (4 μM), rotenone (1 μM) or antimycin A (1 μM). Each data point represents an OCR measurement. **(C)** Individual parameters for basal respiration, proton leak, maximal respiration, respiratory capacity, non-mitochondrial respiration and ATP production were determined. Cells were treated without (Blank control) or with 100 μM H_2_O_2_, 2 mM AKG, or 100 μM H_2_O_2_ plus 2 mM AKG, respectively. Data are expressed as means ± SEM of at least three independent experiments. *P < 0.05 and **P < 0.01.

The effects of H_2_O_2_ and AKG on mitochondrial respiration in IPEC-J2 cells are shown in Figure 6. A general scheme of mitochondrial stress test is shown in Figure 6A. For this section, basal respiration, ATP production, proton leak, maximal respiration, spare respiratory capacity, and non-mitochondrial respiration of treated cell were determined. OCR was the lowest in the H_2_O_2_ group and highest in the AKG group, with intermediate values in the blank control group and the H_2_O_2_ + AKG group, as shown in Figure 6B. Mitochondrial function damage was induced by H_2_O_2_ (100 μM), with a decrease in the basal respiration, proton leak, maximal respiration, spare respiratory capacity, non-mitochondrial respiration, and ATP production (Figure 6C). Conversely, the addition of 2 mM AKG to cells pretreated with 100 μM H_2_O_2_ elevated the rate of mitochondrial respiration when compared with the group treated with 100 μM H_2_O_2_ (*P* < 0.05) (Figure 6C).

### TCA cycle intermediates

**Figure 7:**
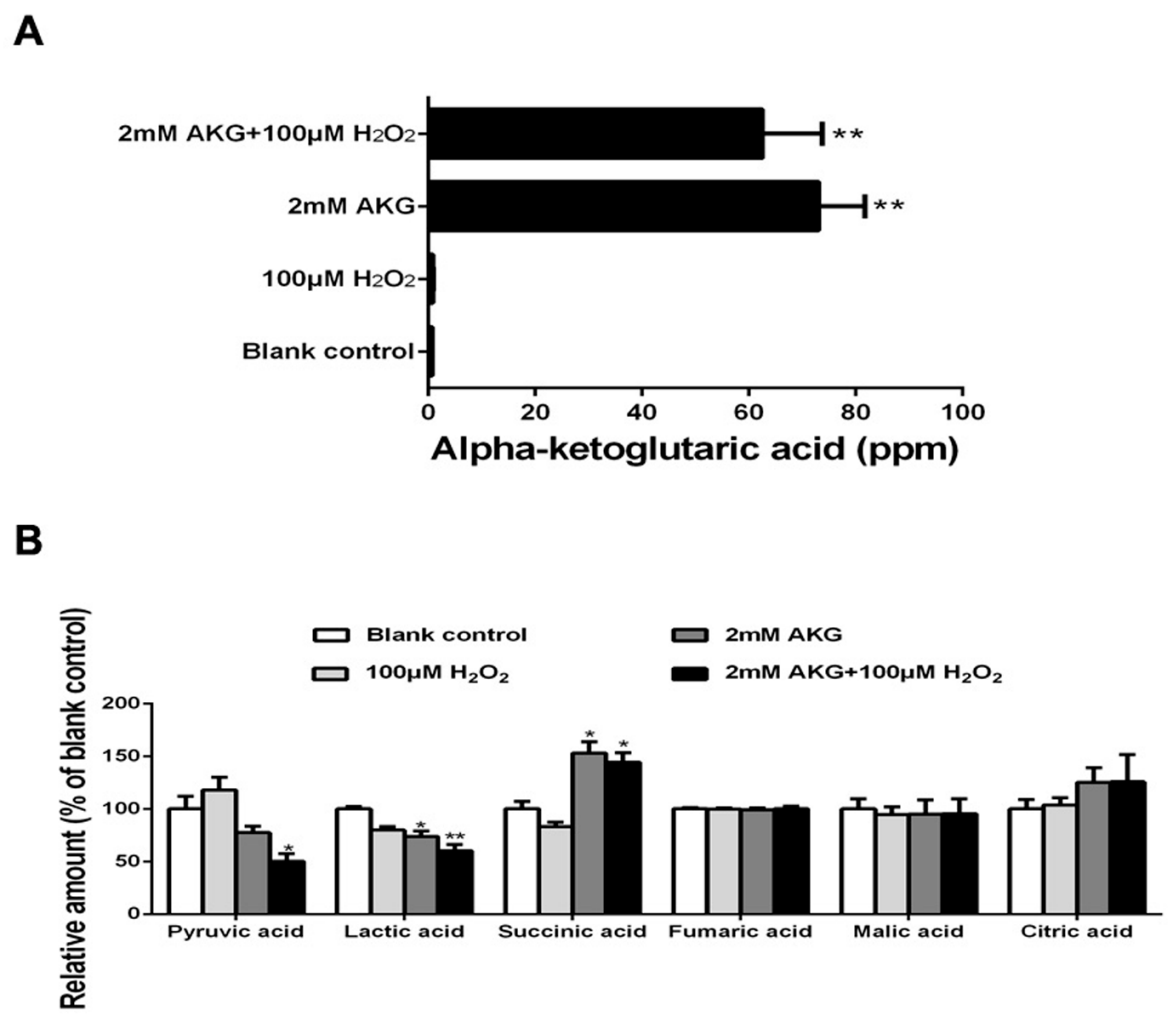
The TCA cycle intermediates, pyruvic acid, and lactic acid of IPEC-J2 cells Cells were treated without (Blank control) or with 100 μM H_2_O_2_, 2 mM AKG, or 100 μM H_2_O_2_ plus 2 mM AKG, respectively. **(A)** Cellular AKG was determined. **(B)** Concentrations of pyruvic acid, lactic acid, and the TCA cycle intermediates (succinic acid, fumaric acid, malic acid, and citric acid) were determined. Data are expressed as means ± SEM of at least three independent experiments. *P < 0.05 and **P < 0.01.

The relative contents of pyruvic acid, lactic acid, and TCA cycle intermediates (citric acid, AKG, succinic acid, fumaric acid, and malic acid) of IPEC-J2 are illustrated in Figure 7. The addition of 2 mM AKG increased the concentrations of succinic acid in the cells (*P* < 0.05), while decreased the concentrations of pyruvic acid and lactic acid (*P* < 0.05). However, there was no difference in fumaric acid, malic acid, and citric acid levels among the groups (*P* > 0.05).

### Antioxidant capacity

**Figure 8:**
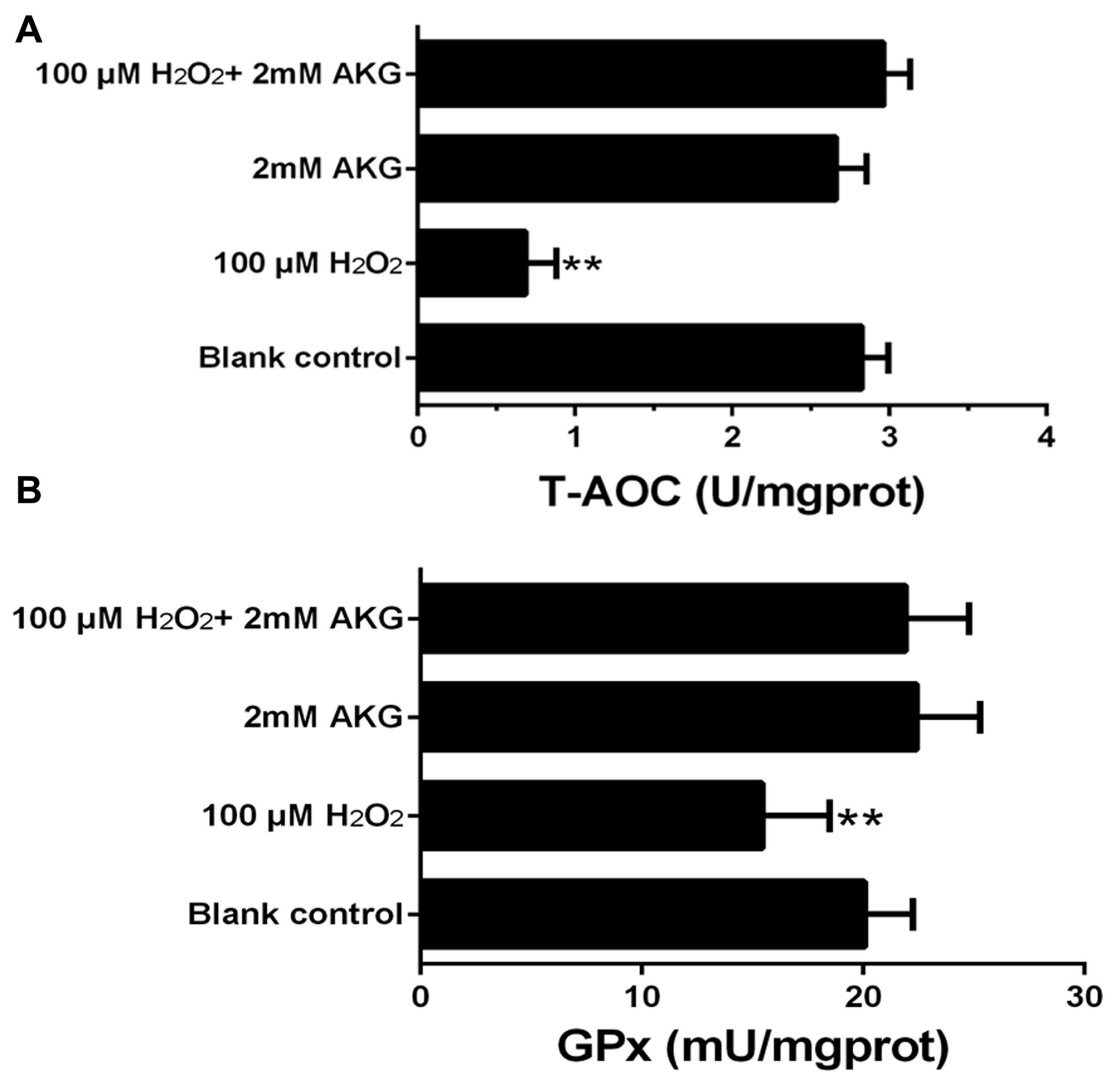
Concentrations of T-AOC and GPx in the IPEC-J2 cells Cells were treated without (Blank control) or with 100 μM H_2_O_2_, 2 mM AKG, or 100 μM H_2_O_2_ plus 2 mM AKG, respectively. **(A)** T-AOC content was determined. **(B)** GPx content was determined. Data are expressed as means ± SEM of at least three independent experiments. *P < 0.05 and **P < 0.01.

The T-AOC and GPx activity analyses are presented in Figure 8. When compared with the blank control, cells in the 100 μM H_2_O_2_ group exhibited a significant decrease in the T-AOC and GPx activities (*P* < 0.05). However, the addition of AKG to cells pretreated with H_2_O_2_ tended to increase these parameters.

### Mitochondrial membrane potential (MMP) and cytochrome c release in cells

**Figure 9:**
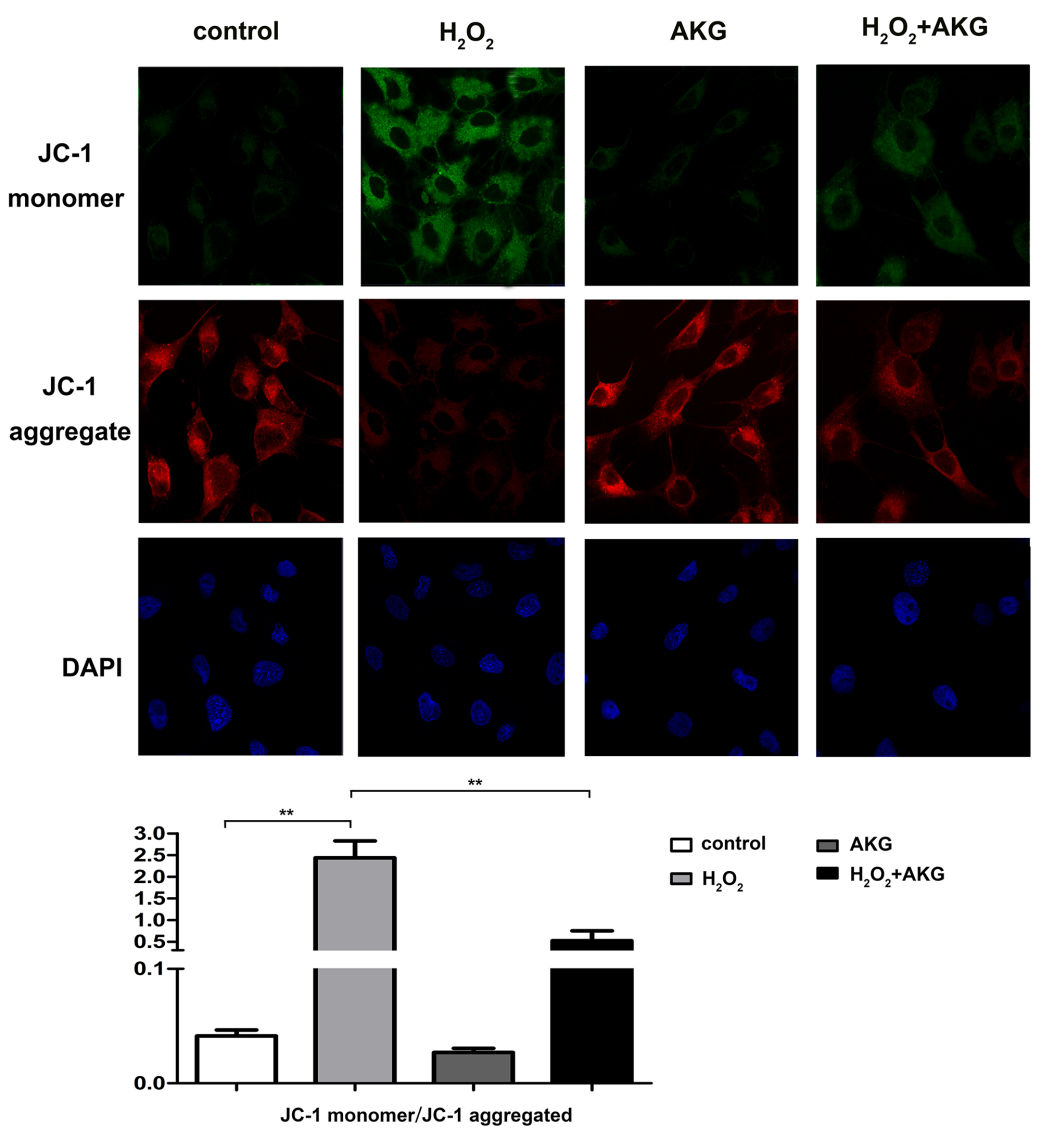
Mitochondrial membrane potential (MMP) of treated cells Cells were treated without (Blank control) or with 100 μM H_2_O_2_, 2 mM AKG, or 100 μM H_2_O_2_ plus 2 mM AKG, respectively. Representative images of JC-1 derived fluorescence in IPEC-J2 with different treatment (400×). The JC-1 monomer was represented with green fluorescence; the JC-1 aggregate image was represented with red fluorescence; the merged images were the combined of the green and red images; The nucleus was stained with DAPI working solution before the photos capture; **(A)** Captured photos are shown. **(B)** Relative ratio of green fluorescence to red fluorescence. Mitochondrial depolarization is indicated by an increase in the green/red fluorescence intensity ratio. Data are expressed as means ± SEM of at least three independent experiments. *P < 0.05 and **P < 0.01.

**Figure 10:**
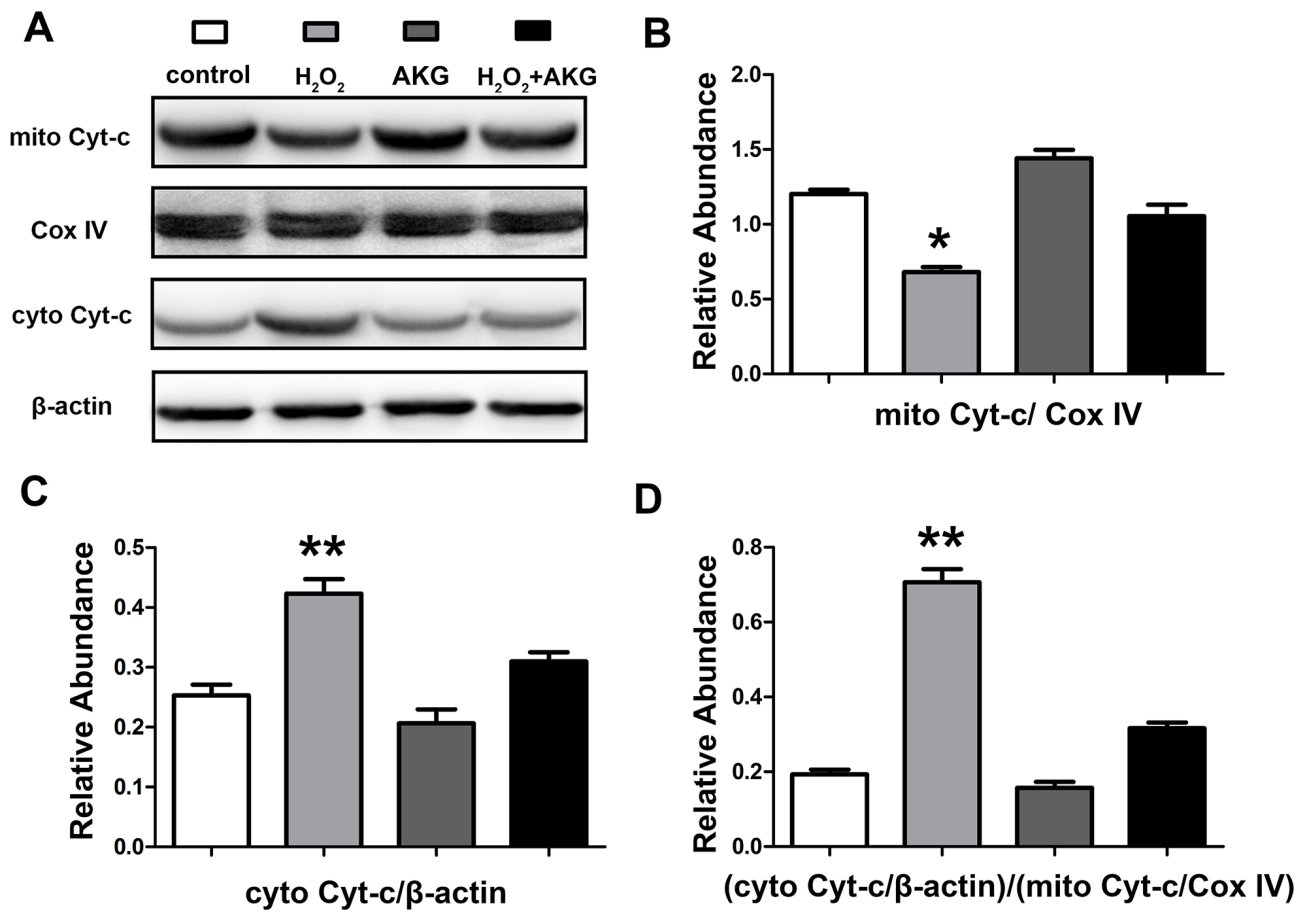
Cytochrome c release of cells After the treatments and mitochondria isolation, protein levels of cytochrome c in the cytoplasm (cyto) and mitochondria (mito) were determined by western blot. β-actin or Cox IV is used as the loading control protein of cytoplasm or mitochondrial. **(A)** Protein bands of mitochondrial cytochrome c, cytoplasmic cytochrome c, β-actin and Cox IV are shown. **(B)** Relative abundance of mitochondrial cytochrome c. **(C)** Relative abundance of cytoplasmic cytochrome c. **(D)** Relative cytochrome c release level was shown. Data are expressed as means ± SEM of at least three independent experiments. *P < 0.05 and **P < 0.01.

To furtherly verify whether alpha-ketoglutarate protected H_2_O_2_-induced apoptosis in IPEC-J2 via a mitochondrial dependent pathway, we determined the profiles of MMP and release of mitochondrial cytochrome c in treated cells. For this JC-1 fluorescence staining, lower intensity of red fluorescence along with the increase of green fluorescence indicate a decrease of MMP and the mitochondrial dependent apoptotic process. Figure 9 showed an increase in green/red fluorescence ratio induced by H_2_O_2_ was counteracted by AKG addition. Treatment with H_2_O_2_ for 4 h may induce the damage of mitochondria, thereby resulting in the release of mitochondrial cytochrome c to the cytoplasm. As shown in Figure 10, the release of cytochrome c was elevated (*P* < 0.05) by H_2_O_2_ treatment and was decreased by AKG. These results indicate that AKG inhibits H_2_O_2_-induced apoptosis via maintaining MMP stabilization, and decreasing the release of mitochondrial cytochrome c.

### Protein abundances of Bcl-2/Bax and caspase-3 activation in treated IPEC-J2

**Figure 11:**
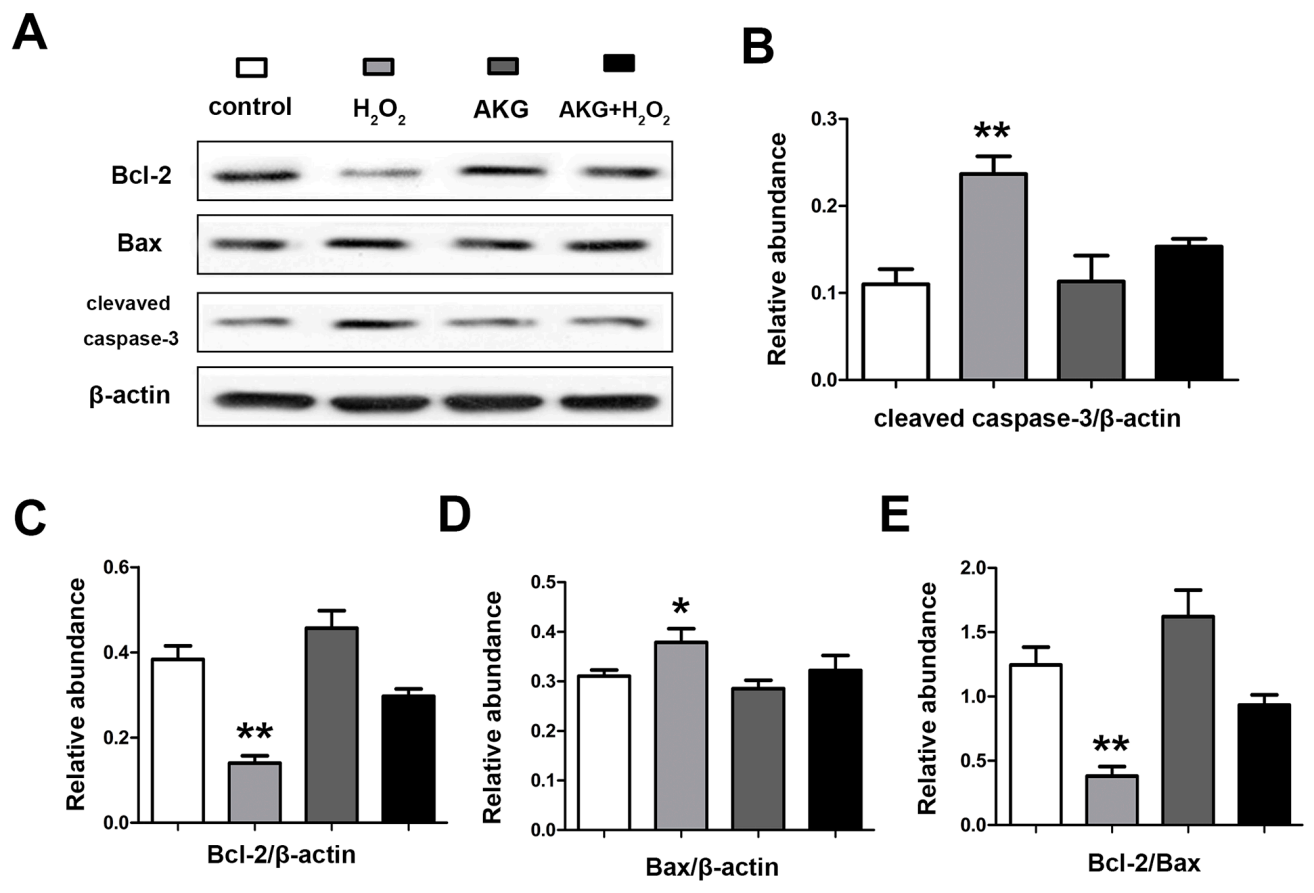
Abundances of apoptosis associated proteins in IPEC-J2 cells determined by western blot analysis Cells were treated without (Blank control) or with 100 μM H_2_O_2_, 2 mM AKG, or 100 μM H_2_O_2_ plus 2 mM AKG, respectively. Protein levels of Bcal-2, Bax, cleaved caspase-3 were determined by western blot. **(A)** Protein bands of Bcl-2, Bax, cleaved capase-3 and β-actin are shown. **(B)** Relative abundance of cleaved caspase-3. **(C)** Relative abundance of Bcl-2. **(D)** Relative abundance of Bax. **(E)** The relative ratio of Bcal-2 to Bax protein is shown. Data are expressed as means ± SEM of at least three independent experiments. *P < 0.05 and **P < 0.01.

In the process of mitochondrial-dependent apoptosis, apoptotic proteins (e.g. caspase-3 and Bax) were activated by the released cytochrome c. The abundances of caspase-3, Bcl-2 and Bax in treated IPEC-J2 cells were determined by western blot. As shown in Figure 11, the ratio of Bcl-2/Bax was the highest in cells treated with 2 mM AKG (*P* < 0.05). The addition of 2 mM AKG to cells pretreated with 100 μM H_2_O_2_ elevated the ratio of Bcl-2/Bax protein when compared with the cells treated with only 100 μM H_2_O_2_ (*P* < 0.05). The abundance of cleaved caspase-3 was highly (*P* < 0.05) upregulated by H_2_O_2_, but significantly (*P* < 0.05) counteracted by AKG.

## DISCUSSION

In swine production, early weaning enhances the serum H_2_O_2_ concentrations in post-weaning piglets, leading to reduced antioxidant capacity and increased free radicals in the body [5]. Although H_2_O_2_ is a weak oxidant and can be easily scavenged by cellular catalase and Gpx, it can severely damage the cultured cells even at lower concentrations [25, 26]. H_2_O_2_ can lead to membrane damage by promoting the release of arachidonic acid from the cell membrane, which may be responsible for the prolonged damage induced by H_2_O_2_ even after being scavenged [27]. Moreover, after rapidly penetrating the cell membrane, H_2_O_2_ can react with intracellular metal ions (such as copper or iron) to generate highly toxic hydroxyl radicals, leading to DNA alterations [25]. Previous studies showed that treatment of SH-SY5Y cells with H_2_O_2_ (400 μM) for 2 h decreased cell survival and viability [28]. In line with these results, our experiments also showed that when IPEC-J2 cells were incubated with H_2_O_2_ (100 μM) for 4 h, cell viability decreased when compared with the blank control. Additionally, the percentages of EdU-positive cells and cells arrested in the S phase significantly reduced in response to the H_2_O_2_ treatment. Moreover, the intracellular ROS content significantly increased in response to 100 μM H_2_O_2_ treatment. These results indicate that H_2_O_2_ induces mitochondrial ROS production, thereby leading to DNA damage in IPEC-J2 cells.

In response to LPS induced damage, DNA cleavage was induced and the proliferation was inhibited by arresting cells in G1/S phase [29]. Increased DNA synthesis may indicate the repair or proliferation of enterocytes for the initial damage [30, 31]. In the present study, an increase in EdU incorporation, cell viability, and cells in the S phase was observed when 2 mM AKG was added to the culture medium. These data indicate that AKG could improve DNA synthesis and promote the progression of 100 μM H_2_O_2_-pretreated IPEC-J2 cells from G0/G1 phase to S phase, ultimately enhancing cell proliferation. The cell cycle is a highly regulated process consisting of three phases, namely, G1/G0, S, and G2/M phase [32]. Cell cycle slows during the G0/G1 phase and does not progress to the S phase until DNA is correctly encoded [33]. Additionally, cell survival and apoptosis are closely related to the cell cycle. Cell apoptosis has periodic characteristics and often occurs during cell cycle arrest. Moreover, apoptosis plays a role in regulating the number of cells. There are potential correlations between apoptosis and cell proliferation [34, 35]. Results of our flow cytometry analysis indicated that 100 μM H_2_O_2_ induced cell apoptosis, while AKG could attenuate this effect. Overall, AKG has the ability to regulate cell proliferation by promoting cell viability and cell cycle progression and concomitantly inhibiting cell apoptosis at both early and late stages.

AKG regulates cellular redox status and plays a role in oxidative defenses [36–38]. Increasing evidence supports this notion. Elevated levels of AKG might confer protection against oxidative damage via its participation in the non-enzymatic oxidative decarboxylation during H_2_O_2_ decomposition [39]. Many studies have reported that AKG inhibits oxidative stress induced by H_2_O_2_ in cultured neurons and erythrocytes [39, 40]. Further evidence showed that AKG improved serum redox homeostasis and conferred protection to arteries against free radical-induced vascular damage. The presence of antioxidant enzymes, such as GPx and superoxide dismutase, prevented the oxidative damage in cells [16]. Consistent with our *in vitro* mechanistic studies, dietary supplementation of AKG (1%) ameliorated intestinal injury caused by oxidative damage in LPS-challenged piglets [12]. In line with these observations, our study also found that H_2_O_2_ exposure induced oxidative stress, as evidenced by decreases in cell survival and viability, while AKG treatment markedly improved the antioxidant function. Thus, AKG may protect porcine small intestinal epithelial cells against oxidative injury via the induction of antioxidant cellular defenses.

Endogenous ROS is mainly produced by the respiratory chain of the mitochondria [6]. In our previous report, mitochondrial dysfunction was observed with a decrease in basal respiration, maximal respiration, and non-mitochondrial respiration after LPS treatment [1]. Our current results support this notion by providing data on basal respiration, maximal respiration, respiratory capacity, non-mitochondrial respiration, and ATP production in IPEC-J2 cells. The mitochondrion is responsible for the generating ATP, maintaining biological functions and meeting higher demands for energy when cell responded to various stress [41]. In this regard, our current finding reveals AKG enhanced mitochondrial bioenergetics, when cell responded to oxidative stress.

Consisted with the positive effect of AKG on mitochondrial bioenergetics, it is evident from our study that an increase in AKG concentration can stimulate the TCA cycle. AKG is an intermediate of the TCA cycle and may affect ATP production [42]. We further determined the content of TCA cycle intermediates (citric acid, AKG, succinic acid, fumaric acid, and malic acid), as well as those of pyruvic acid and lactic acid pathways, in IPEC-J2 cells after AKG treatment. Our results showed that AKG treatment increased cellular concentrations of succinic acid. Previous studies have demonstrated an increase in ROS production at sub-saturating succinate concentrations (0–0.1 mM), while a decline in ROS production was observed at higher succinate concentrations (0.1–5.0 mM) [43]. Other TCA cycle intermediates, namely, fumarate, malate, citrate, and, especially, oxaloacetate that binds competitively to the dicarboxylate binding site of complex II also attenuated ROS production [43]. Additionally, succinate provides higher yields of ATP in a dose-dependent manner, (0-15mM) [44]. Along with these previous reports, our results show that AKG treatment increases the concentration of TCA cycle intermediates, especially succinic acid, contributing to mitochondrial redox homeostasis and ATP production. Thus, we speculate that AKG may regulate the mitochondrial respiratory chain via the TCA cycle intermediates.

Furthermore, AKG supplementation increased the T-AOC and GPx in H_2_O_2_-treated IPEC-J2 cells. The T-AOC is an integrative index used to reflect the antioxidant capacity of the cells [36]. Collectively, our results indicated that AKG treatment resulted in decreased enterocyte apoptosis with a concomitant increase in antioxidant ability. Therefore, AKG may attenuate the cytotoxicity by maintaining the balance of ROS in stress responsive cells.

The extrinsic death receptor-mediated pathway and the intrinsic mitochondrial pathway have been known as the major signal pathways involved in the apoptotic process [45]. The apoptotic process is composed of a proteolytic cascade involving a family of caspase proteases [46]. Expression of Bcl-2 and Bax in cells is usually associated with this apoptosis process. [47]. Increased level of Bax leads to the combination of Bcl-2, low concentration of Bcl-2 in the cell, and activation of cell apoptosis [48]. During the apoptotic process, Bax forms oligomers and transfers from cytoplasm to mitochondrial membrane [49], then Bax oligomer interacts with pore protein of mitochondrial membrane, results in increased membrane permeability, and induces release of cytochrome c from mitochondria to cytosol, finally actives the caspase [50], leading to mitochondrial electron transport, DNA fracture and furtherly deterioration, thereby caused apoptosis [51]. To furtherly verify the anti-apoptotic mechanism of alpha-ketoglutarate, MMP and apoptotic proteins of mitochondrial pathway were determined. In this study, it was demonstrated that AKG treatment increased Bcl-2/Bax ratio and MMP, and decreased cytochrome c release from mitochondria into the cytoplasm when intestinal cell responded to H_2_O_2_-induced stress milieu. Combining with the results described in early part, we proposed that mitochondria-dependent pathway is involved in the anti-apoptosis effect of AKG on oxidative stress-responsive intestinal cells.

In summary, H_2_O_2_ induced mitochondrial dysfunction and cell-cycle impairment, while AKG promoted DNA synthesis and mitochondrial bioenergetics in intestinal epithelial cells. Further results suggest that this protective effect partly attributes to the attenuation of mitochondrial-dependent apoptosis. Possible anti-apoptotic mechanisms involve the following steps: improvements in TCA cycle and ATP production, decreased cellular ROS content, enhanced the expression of Bcl-2, stabilization of MMP, decreased release of mitochondrial cytochrome c, and inhibition of caspase-3 activation. These novel results provide reference to support the use of AKG as a potential nutrient on attenuating some disease symptoms induced by mitochondrial dysfunction or oxidative stress.

## MATERIALS AND METHODS

### Reagents

Dulbecco’s modified Eagle’s medium, high glucose (DMEM-H), fetal bovine serum (FBS), and antibiotics were purchased from Invitrogen (Grand Island, NY, USA). Plastic culture plates were manufactured by Corning Inc. (Corning, NY, USA). Unless indicated, all other chemicals were purchased from Sigma-Aldrich (St. Louis, MO, USA).

### Cell culture

IPEC-J2 cells were cultured in the plastic culture flasks (25 cm^2^) with DMEM-H containing 10% FBS, 5 mM L-glutamine, 100 U/mL penicillin, and 100 μg/mL streptomycin. At reached to 80% confluence, cells were trypsinized and seeded in 6-well culture plates with 8 × 10^3^ cells per well and kept in a 5% CO_2_ humidified incubator at 37 °C. After overnight incubation, the culture medium was replaced by basal medium (blank control, groups 1 and 3) and basal medium + 100 μM H_2_O_2_ (groups 2 and 4) for 4 h. Thereafter, 2 mM AKG was added to groups 3 and 4, and the medium was changed as described above. The cells were collected for subsequent analysis after culturing for 2 days.

### Cell viability assay

Cell counting kit-8 (CCK-8, Dojindo, JPN) was used to determine the cell viability, according to the protocol. Initially, IPEC-J2 cells were incubated in media containing different treatments. Then, the culture media were replaced with 100 μL of fresh media containing 10 μL reagent from the kit. After incubation for 45 min at 37 °C, the absorbance of each well at 450 nm was determined with an ELISA plate reader (Bio-Tek, Winooski, VT, USA). The results of cell viability were shown as OD_450_ values. The optimum concentration of H_2_O_2_ or AKG obtained from the cell viability assay was used in further analyses.

### Measurement of DNA synthesis

After the treatments, DNA synthesis rate of the treated cells was measured by incorporating 5-ethynyl-2′-deoxyuridine (Invitrogen, USA) Cell-light™ EdU Kit (Rui Bo Biotechnology, CHN), as described in our previous studies [1]. After the incubation process of EdU staining according to the product manual, an Olympus B × 51 microscope (Olympus, JPN) was used to capture the image of EdU-positive cells counter-stained with Apollo® 567 Hoechst 33342. Five more different microscopic fields were randomly selected for the capture at 200× magnification.

### Profiles of the cell cycle and apoptosis analyzed by flow cytometry

About 1 × 10^6^ treated cells were collected and centrifuged at 1 000 ×*g* for 5 min. The supernatant was discarded, then washed cells once with ice-cold PBS and resuspended in 1 mL of the staining reagent containing 50 mg/mL propidium iodide (PI) and 100 mg/mL RNase for 30 min in the dark. To assess apoptosis, the pretreated cells were stained with PI/Annexin-V-FITC (KeyGEN, CHN) according to the manufacturer’s instructions. Cell cycle arrest and apoptosis were analyzed by flow cytometry (BD FACSCalibur, USA). Fluorescence intensities of cells stained with PI and Annexin-V-FITC were monitored at 630 and 525 nm, respectively.

### Metabolic assays

The effects of AKG addition on mitochondrial respiration in H_2_O_2_-induced cells was determined by an XF-24 Extracellular Flux Analyzer and a Cell Mito Stress Test Kit (Seahorse Biosciences, USA), as described previously [1]. Briefly, after the treatment, the culture medium was replaced by the specific test medium. During the process of test, the specific concentration of oligomycin, carbonyl cyanide-p-trifluoromethoxyphenylhydrazone (FCCP) as well as rotenone and antimycin A were regularly and automatic injected into the test medium. ATP production, proton leak, basic respiration and maximal respiration capacity was assessed by the data obtained from the specific probes. In addition, the cellular total protein was determined for the final normalization.

### Mitochondrial membrane potential determination

A kit of membrane permeability dye JC-1 (Beyotime, CHN) was used for this test. JC-1 is an indicator of mitochondrial membrane potential loss. The dye of JC-1 enters the mitochondria of normal cells, emits red fluorescence, and the dye enters the cytoplasm of apoptotic cells as a monomer that emits green fluorescence. Fluorescence measurement using a confocal microscopy (Leica, GER). The proportion of green fluorescence and red fluorescence showed the change of mitochondrial membrane potential. Five more different microscopic fields were randomly selected for the capture.

### Mitochondria isolation

Mitochondria were isolated from cultured cells using Mitochondria Isolation Kit for Cultured Cells (TransGen Biotech, CHN). For the isolation, about 1 x 10^7^ cells per sample were used. During the process, 800 μL of mitochondrial separation reagent A, 10 μL mitochondrial separation reagent B and 500 μL mitochondrial separation reagent C was used, the specific procedures were strictly abided by the protocol manual. In addition, all the operation was kept on the ice, and protease inhibitor and phosphatase inhibitors were extra added into the regents before the isolation process. Cytoplasmic and mitochondrial fractions were separated for western blot analyze. Cox IV (Abcam, Cambridge, UK) was used as a loading control for mitochondrial proteins.

### ROS analysis by flow cytometry

Flow cytometry was used for intracellular ROS level determination, as described in our previous study [52]. Briefly, after the indicated treatments, IPEC-J2 cells were incubated with 20 μM 2,7-dichlorodihydroflurescein diacetate for 30 min at 37 °C and washed twice with PBS. Fluorescence intensities of 10,000 cells were analyzed using CELLQuest™ software in FACScalibur™ (Becton Dickinson, San Jose, CA).

### Detection of TCA cycle intermediates by GC-MS

Treated cells were fixed for detection of metabolites by GC-MS, the procedure was referred as its described previously [53]. The cells were washed with PBS twice and treated with 0.25% trypsin. They were then centrifuged at 1000 ×*g* for 5 min. After being quenched by 500 μL of pre-chilled 50% (v/v) methanol, cells were centrifuged again at 1000 ×*g* for 5 min and the supernatant was discarded, followed by addition of 500 μL of pre-chilled 100% (v/v) methanol. The metabolites were detected using an Agilent 7890B-5977A GC-MS equipped with a HP-5ms (30m × 250μm × 0.25 μm) capillary column (Agilent J&W, Santa Clara, CA, USA). All metabolites were previously validated using authentic standards (Sigma).

### Detection of anti-oxidant capacity

Total antioxidant capacity (T-AOC) and glutathione peroxidase (GPx) assay kits (Nanjing Jiancheng, CHN) were used for the determination. The procedures strictly abided by the manufacturer’s instructions. A UV/visible spectrophotometer-UV-2450 (SHIMADZU, Kyoto, Japan) was used for the data output.

### Western blotting analysis

After the treatments, cells or isolated mitochondrial fractions were lysed in RIPA buffer (Beyotime, CHN), along with the protease inhibitor and phosphatase inhibitors. Protein concentrations of the lysis were measured BCA kit (Beyotime, CHN), as described in a previous study [31]. All samples were diluted to an equal protein concentration in each sample. Samples of protein lysis were high-temperature pre-denaturized and subjected to SDS-PAGE, then transferred to polyvinylidene difluoride membranes, which were blotted with 5% nonfat milk in TBS-with 0.05% Tween-20 for 1 h and incubated overnight with a primary antibody, followed by horseradish peroxidase-linked secondary antibody. The protein bands were visualized using chemiluminescence reagents. The densities of the protein bands were determined by Imager 2200 software (Alpha Innotech Corporation), and the relative level of target proteins was shown as the density percentage of the control loading protein (e.g. β-actin, Cox IV).

### Statistical analysis

All results are expressed as mean ± SEM. The statistical analysis was performed using one-way ANOVA, SAS 8.2. Differences among group means were compared using the Duncan multiple comparison tests. Probability values less than 0.05 were considered as statistically significant. All the experiments were repeated independently at least three times.
